# Effects of rocky desertification on soil bacterial community in alpine grasslands of the Qinghai-Tibet Plateau

**DOI:** 10.3389/fmicb.2024.1485069

**Published:** 2025-01-06

**Authors:** Shan Li, Huakun Zhou, Wenying Wang, Haze Ade, Zhonghua Zhang, Li Ma, Zhen Wang, Qiang Zhang, Jingjing Wei, Hongye Su, Ruimin Qin, Zhengchen Shi, Xue Hu, Faliang Wu

**Affiliations:** ^1^College of Geographical Sciences, Qinghai Normal University, Xining, China; ^2^Qinghai Provincial Key Laboratory of Restoration Ecology in Cold Regions, Northwest Institute of Plateau Biology, Chinese Academy of Sciences, Xining, China; ^3^College of Life Sciences, Qinghai Normal University, Xining, China; ^4^University of Chinese Academy of Sciences, Beijing, China; ^5^College of Agriculture and Animal Husbandry, Qinghai University, Xining, China

**Keywords:** Qinghai-Tibet Plateau, alpine grassland, rocky desertification, soil properties, soil bacterial community

## Abstract

The makeup of soil microbial communities may serve as a crucial predictor of the alpine grassland ecosystem. Climate change and human disturbance have resulted in intensified ecosystem degradation, such as grassland rocky desertification, which may modify the structures and composition of the microorganisms. However, little is known about the effects of rocky desertification on soil microbial communities of soil. Here, we investigated five different layers of rocky desertification grassland in the Qinghai-Tibet Plateau, including nil rock desertification (NRD); potential rocky desertification (PRD); light rocky desertification (LRD); moderate rocky desertification (MRD); and severe rocky desertification (SRD), we compared soil bacterial community with soil physiochemical properties in different rocky desertification conditions. The result showed that rocky desertification significantly altered the physiochemical properties of the soil but did not significantly affect the bacterial community microbial abundance and diversity. At the same time as rocky desertification increased, soil organic carbon (SOC), total nitrogen (TN), alkali hydrolyzable nitrogen (AN), available phosphorus (AP), and available potassium (AK) decreased significantly, while soil pH, total phosphorus (TP); and total potassium (TK) increased. Redundancy analysis revealed that pH, AK, TP, and SOC are key factors influencing soil bacterial communities. Our finding provides basic information and scientific reference for the restoration of the rocky desertification of alpine grasslands.

## 1 Introduction

Grasslands are an important component of global terrestrial ecosystems, covering nearly 40% of the earth’s land area (excluding permanent ice cover areas), and play a key part in regulating climate change by balancing greenhouse gases (Scurlock and Hall, 1998). Alpine grassland is one of the most important grassland types, with more than 48% of alpine grassland occurring on the Qinghai-Tibetan Plateau (Lin et al., 2015). In the last few decades, as a result of climate change, overgrazing, and other factors (degradation of permafrost and damage by rodents, etc.), alpine grasslands have extensively degraded, and land productivity gradually weakened, seriously affecting the normal operation of alpine grassland ecosystem services (Lü et al., 2016; Parras-Alcántara et al., 2015; Zhang et al., 2017). The Tibetan Plateau region, as a sensitive area to global climate change, is experiencing grassland desertification according to the team’s latest investigations, making it an ideal area to study desertification of fragile ecosystems in light of climate change and human activities.

Desertification is a widespread issue worldwide and poses a severe threat to the ecological environment. This is evidenced by a decline in forest cover, with forests degrading into shrubs, grasslands, or even bare land, a reduction in plant species, and a move toward a more simplified community structure (Jiang et al., 2014; Prãvãlie, 2016). Typically, desertification occurs in arid and semi-arid regions (Prãvãlie et al., 2019). Rocky desertification is a typical land degradation phenomenon and one of the most threatening environmental problems in limestone areas, the causes of which include severe soil erosion, large-scale exposure of basement rocks, drastic decline in productivity, and the emergence of desert-like landscapes as a result of irrational land use on the fragile karst eco-environment (Sheng et al., 2015; Sweeting, 1995; Wang K. et al., 2019). The intricate processes such as frost heaving and thawing of glacier snow occur on the Qinghai-Tibet Plateau of China. These processes destroy the soil structure, disrupt energy and material transfer, offer material conditions for wind and water erosion, and eventually form rock desertification landscapes. Yet, there are few studies on the rocky desertification of the Qinghai-Tibet Plateau.

Soil microorganisms play key roles in the Earth’s elemental cycles (Luo et al., 2017; Zhou et al., 2019; Wei et al., 2023) and are essential for ecological processes such as soil biomass decomposition and nutrient cycling (Delgado-Baquerizo et al., 2016; Chen et al., 2016). These microorganisms are highly sensitive to external disturbances, with factors such as plant composition, biomass, pH, and soil nutrients significantly influencing microbial community structure (Li et al., 2021; Wang C. et al., 2019; Zhang et al., 2022; Zheng et al., 2023). Additionally, the availability of trace metals, mineral or rock types, and their chemical compositions also play critical roles in shaping microbial community composition. Trace metals act as essential cofactors for enzymes involved in microbial metabolic processes, thereby influencing community structure and function (Dong et al., 2022). Furthermore, the mineralogical and geochemical properties of rocks and minerals can modulate nutrient availability and microbial colonization patterns, shaping the distribution and interactions of microbial communities (Sheng et al., 2023). In areas of rocky desertification, soil bacteria play an important role, not only as core members of the soil microbial community but also as key to maintaining soil quality, supporting vegetation growth, and promoting ecosystem recovery. Environmental factors, especially changes in precipitation and temperature, have been shown to affect soil bacterial communities significantly. In addition, the degree of rocky desertification influences the composition of soil bacterial communities, while the release of bacterial extracellular enzymes influences the biomass, community dominance and relative abundance of soil bacteria. These factors shape the abundance of soil bacteria and their interactions with aboveground plant communities (Shen et al., 2013; Chen et al., 2018; Xiao et al., 2021; Wu et al., 2023). As rocky desertification deepens, the composition and diversity of soil microbial communities change in response to changes in soil nutrient effectiveness. Understanding the response of microbial communities to rocky desertification and the factors influencing them can provide important information for grassland health assessment and management.

The main objective of this study was to examine the changing patterns and regulatory factors of soil bacterial communities during the rocky desertification of alpine grasslands. In Qinghai-Tibet Plateau, we studied the effects across five rocky desertification levels, nil rock desertification (NRD), potential rocky desertification (PRD), light rocky desertification (LRD), moderate rocky desertification (MRD), and severe rocky desertification (SRD). Our objectives were to: (1) How different extents of rocky desertification affect soil bacterial community changes, and (2) Which soil variables are primarily responsible for changes in soil bacterial community structure and diversity.

## 2 Materials and methods

### 2.1 Study site sampling collection

The study area was the alpine grasslands of Guoluo Prefecture on the hinterland of the Qinghai-Tibet Plateau and the source of the Yellow River (97°54′∼ 120°50′E, 32°31′∼35°40′N). Its average altitude is from 3,500 to 5,000 m. The research area has a typical continental plateau climate, with an average annual temperature of −4.5°C and 400–500 mm of precipitation that increases from west to east. The plants grow slowly, in fragile, and sensitive ecosystems. Under the combined influence of global climate variability and human activities, glaciers on the Qinghai-Tibet Plateau retreat, snow melt, and animal trampling destroy the structure of the alpine grasslands, accelerate soil erosion, expose large areas of bedrock, and cause grassland degradation in the form of a landscape of rocky desertification.

**FIGURE 1:**
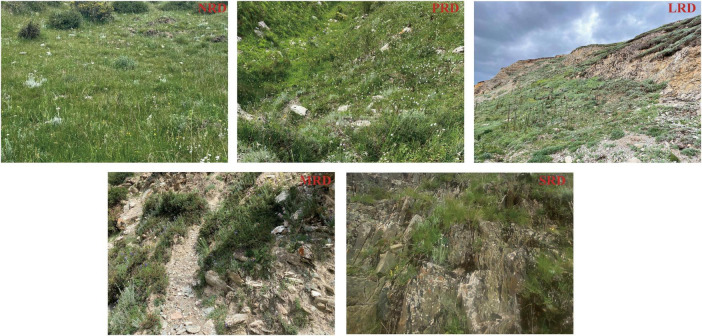
Pictures of the differently degraded rocky desertification. NRD, nil rock desertification; PRD, potential rocky desertification; LRD, light rocky desertification; MRD, moderate rocky desertification; SRD, severe rocky desertification.

We selected five different types of sites for this study, based on the rock cover of the soil surface determined by visual inspection. The rocky desertification of the alpine Qinghai-Tibet grasslands has not been classified. Therefore, the classification of rocky desertification was based on the team field investigation and a study by Xiong et al. (2002) who proposed that kart rocky desertification is categorized based on four factors (slope, percentage of vegetation, percentage of bare rock, and average depth of surface soil). Therefore, considering the locational characteristics of the study area, we selected five sample plots representing zero rock desertification (NRD), potential rock desertification (PRD), light rock desertification (LRD), moderate rock desertification (MRD), and severe rock desertification (SRD) based on vegetation cover, soil depth, exposed rock, and grazing disturbance (Table 1). Among these, NRD exhibits the highest vegetation cover and soil depth, while SRD is characterized by the lowest vegetation cover and significant bedrock exposure. MRD represents the intermediate stage, where the balance between vegetation and exposed rock is more pronounced, and the soil is thinner (Figure 1). The threshold of each degree is listed in Table 1. For simplicity, the sample plots were abbreviated as NRD, PRD, LRD, MRD, and SRD in the following context.

**TABLE 1:** Basic information and classification standard for different grades of rocky desertification in the study.

Sample plot	Longitude, latitude	Altitude/m	Slope/°	Percentage of plant/%	Percentage of bare rock/%	Average depth of topsoil/cm	Interference conditions
NRD	34°37′27″ 100°33′16″	3,506	< 15	> 80	< 20	> 30	Light grazing disturbance
PRD	34°42′52″ 100°44′18″	3,581	> 15	< 80	> 20	< 30	Light grazing disturbance
LRD	34°31′50″ 100°26′45″	3,750	> 15	< 70	> 40	< 20	Grazing disturbance
MRD	34°34′50″ 100°33′26″	3,417	> 20	< 50	> 50	< 15	Grazing disturbance
SRD	35°30′23″ 100°49′16″	3,649	> 25	< 30	> 70	< 10	Without disturbance

NRD, nil rock desertification; PRD, potential rocky desertification; LRD, light rocky desertification; MRD, moderate rocky desertification; SRD, severe rocky desertification.

In mid-July 2023, different rocky desertification types were selected as experimental sample plots in the study area, and three sample squares measuring 5 m × 5 m were randomly laid out in each plot, with a certain interval between sample plots. Within each sample square, 6 sampling points were selected along the diagonal, the surface cover was removed, and soil samples at the surface layer (0–15 cm) were collected using a 70 mm diameter soil auger. To avoid spatial heterogeneity, the soil samples from the six sampling points were uniformly mixed into one replicate. Collected soil samples were mixed and passed through a sieve (sieve holes less than 2 mm) to remove plant material, then stored in sterilized, self-sealing bags and promptly returned to the laboratory. One part of the samples were collected in 15 ml sterile centrifuge tubes and placed in a freezer at −80°C to determine 16SrRNA; the other part was placed in a ventilated environment for determination of the physiochemical properties of the soil.

### 2.2 Determination of soil physical properties

Soil pH was determined by preparing a mixture of soil and water at a ratio of 1:2.5 (soil to water). The pH of this mixture was then measured using a pH conductivity meter (Multi-Parameter PC TestrTM 35, Japan). The sample’s SOC was determined using the heating procedure with potassium dichromate sulfuric acid. The Kjeldahl technique method was used to determine total soil nitrogen (TN) (Xie et al., 2015). After the sample was wet digested with H2SO4 and HClO4, the total phosphorus (TP) was measured colorimetrically using an ultraviolet spectrophotometer (UV2800A, Shanghai, China). Using an FP6431 flame photometer (Shanghai, China), flame emission spectroscopy was used to determine the soil’s total potassium (TK) level. Alkali’s hydrolyzable nitrogen (AN) was calculated using the decomposed distillation technique (Fu et al., 2004). The molybdenum blue technique was used to assess the accessible phosphorus (AP) concentration (Xiong et al., 2015). The ammonium acetate–flame photometer technique was used to assess available potassium (AK) (Wu et al., 2014).

### 2.3 Soil DNA extraction and sequencing

Total soil DNA was extracted using EZNa^®^ soil DNA kits from OMEGA (United States), and the extracted product was confirmed by 1% agarose gel electrophoresis. The hypervariable region V3-V4 of the bacterial 16S rRNA gene was amplified using the primer pairs 338F (5′-ACTCCTACGGGAGGCAGCAG-3′) and 806R (5′-GGACTACH VGGGTWTCTAAT-3′) on a T100 Thermal Cycler PCR thermocycler from BIO-RAD (USA). Following an initial denaturation at 95°C for three minutes, there were 27 cycles of denaturation at 95°C for thirty seconds, annealing at 55°C for thirty seconds, and extension at 72°C for forty-five seconds. The final extension was kept at 4°C for ten minutes. Following the manufacturer’s instructions, the PCR product was extracted from a 2% agarose gel and purified using the YuHua (Shanghai, China) PCR Clean-Up Kit. Thermo Fisher Scientific’s (USA) Qubit 4.0 Fluorometer was used to measure the concentration of the purified DNA.

The raw FASTQ files were demultiplexed using an internal Perl script. Subsequently, they underwent quality filtering by FASTP version 0.19.6 and were merged using FLASH version 1.2.7.

(i) Truncating reads with an average quality score < 20 over a 50 bp sliding window, discarding those < 50 bp and reads with ambiguous characters; (ii) Assembling overlapping sequences (> 10 bp overlap, ≤ 0.2 maximum mismatch ratio); discarding unassimilable reads; (iii) Distinguishing samples by barcode and primer, modifying the direction of the sequence when precise barcode matching is achieved, and permitting two nucleotide mismatches when matching primers. After that, at a 97% sequence similarity level, the optimized sequences were grouped into operational taxonomic units (OTUs) using UPARSE 7.1. Each OTU’s most prevalent sequence was selected to serve as the representative sequence. Chloroplast sequences from each sample were manually removed to filter the OTU table. The average Good coverage of 99.09% was maintained by rarefying the number of 16S rRNA gene sequences per sample to reduce the effect of sequencing depth on the alpha and beta diversity metric.

### 2.4 Statistical analysis

One-way ANOVA was used in SPSS 16.0 software to examine the parameters of the vegetation and soil, and Duncan’s tests (*P* < 0.05) were used to determine statistical significance. Major Bio-Cloud^1^ was used for the bioinformatic study of soil microbiota. Mothur v1.30 was used to construct OTU information, rarefaction curves, and alpha diversity indices (which include observed OTUs, Chao1 richness, Shannon index, and Good’s coverage). Using the Vegan v2.5-3 package in R Studio, principal coordinate analysis (PCoA) based on Bray-Curtis dissimilarity was used to evaluate the similarity across microbial communities across samples. Bacterial communities at different levels of rock desertification were identified using the linear discriminant analysis of effect size (LEfSe) method.^2^ To exclude environmental components with a VIF larger than 10, variance inflation factors (VIF) were computed for each environmental variable. The Vegan v2.5-3 software was used to perform distance-based redundancy analysis (db-RDA) in order to evaluate the association between the environmental conditions and the makeup of the soil bacterial community. Monte Carlo permutation tests were used as the basis for forward selection. The relationships between the relative abundance of soil bacterial communities and environmental variables were examined using the Spearman correlation matrix. The varpart function of the vegan package in R Studio was used to conduct variance partitioning analysis (VPA) in order to ascertain the relative significance of plant and soil variables in influencing the makeup of the soil bacterial population.

## 3 Results

### 3.1 Soil physicochemical characteristics

**FIGURE 2:**
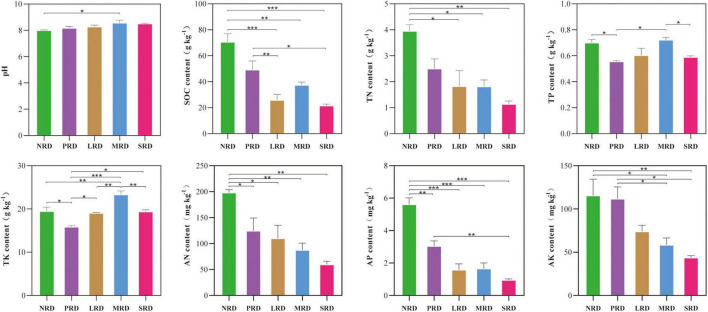
The soil properties of the differently degraded rocky desertification (*n* = 3). NRD, nil rock desertification; PRD, potential rocky desertification; LRD, light rocky desertification; MRD, moderate rocky desertification; SRD, severe rocky desertification; SOC, soil organic carbon; TN, total nitrogen; TP, total phosphorus; TK, total potassium; AN, alkali hydrolyzable nitrogen; AP, available phosphorus; AK, available potassium. *P ≤ 0.05, **P ≤ 0.01, ***P ≤ 0.001.

The study found significant changes (*P* < 0.05) in soil pH and the contents of SOC, TN, TP, TK, AN, AP, and AK across varying degrees of rocky desertification (Figure 2). Soil pH increased gradually with more severe desertification, becoming weakly alkaline, and also increased with depth. Significant pH differences were noted between nil rock desertification (NRD) and moderately rocky desertified (MRD) soils. SOC content varied significantly among desertification levels, being highest in NRD and lowest in severely rocky desertified (SRD), with significant differences compared to lightly (LRD), moderately (MRD), and SRD (*P* < 0.01). TN content followed a pattern: NRD > PRD > LRD > MRD > SRD, with NRD significantly higher than SRD (*P* < 0.01). Both SOC and TN tended to decrease with soil depth. TP and TK exhibited similar trends, both reaching their highest levels in MRD and lowest levels in PRD. The TP content showed a significant difference between the two groups (*P* < 0.05), while the TK content displayed a highly significant difference between PRD and MRD (*P* < 0.001). The content of AN exhibited a decreasing trend with the intensification of rocky desertification, reaching its highest level in NRD and lowest in SRD, with significant differences observed between these two levels (*P* < 0.05). Similarly, AK content followed a comparable decreasing pattern, peaking in NRD and dropping to its lowest in SRD. These differences were statistically significant across the desertification levels (*P* < 0.01). AP showed a variation rule: NRD > PRD > MRD > LRD > SRD, with extremely significant differences compared to LRD, MRD, and SRD (*P* < 0.001).

### 3.2 Soil bacterial community structure

**FIGURE 3:**
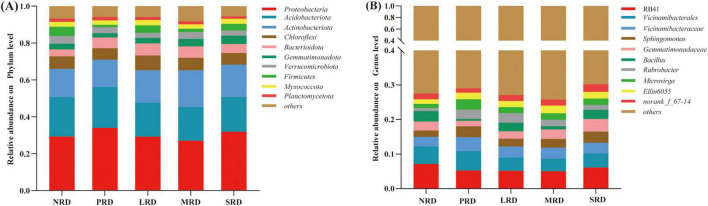
The relative abundance of the phylum **(A)** and genus **(B)** dominating bacterial populations under varying degrees of rocky desertification. NRD, nil rock desertification; PRD, potential rocky desertification; LRD, light rocky desertification; MRD, moderate rocky desertification; SRD, severe rocky desertification.

The microbial composition at the phylum and genus level is shown in Figure 3. Proteobacteria, Acidobacteria, Actinobacteria, Chloroflexi, Bacteroidota, Gemmatimonadetes, Verrucomicrobia, Firmicutes, Myxococcota, Planctomycetota, etc. were the predominant phylum of the soil bacterial community in all samples. Proteobacteria was the most abundant across all samples, and the average abundance was 29.28, 33.99, 29.20, 26.99, and 31.84% in NRD, PRD, LRD, MRD and SRD, respectively. The second most dominant phylum was Acidobacteria with an average abundance of 21.29, 22.08, 18.42, 18.25, and 18.82% in NRD, PRD, LRD, MRD and SRD, respectively. Following Acidobacteria, the phyla were dominant by Actinobacteria and Chloroflexi, the relative abundance of Actinobacteria and Chloroflexi decreased and then increased with the intensification of rocky desertification (Figure 3A). The relative abundance of RB41, Vicinamibacterales, Sphingomonas, and Gemmatimonadacese first fell and then grew at the genus level of the bacterial population. As rocky desertification deepened, the relative abundance of Vicinamibacteraceae steadily declined (Figure 3B).

### 3.3 Soil bacterial community diversity

**FIGURE 4:**
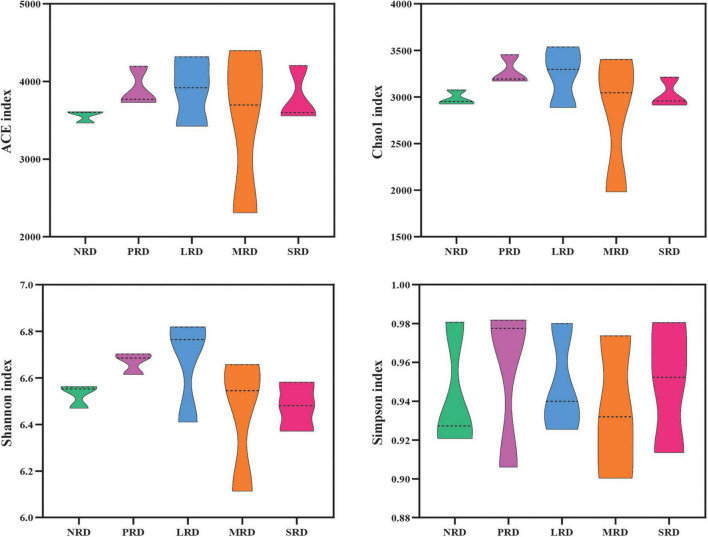
The features of the richness and diversity indices of soil bacteria at varying degrees of rocky desertification. NRD, nil rock desertification; PRD, potential rocky desertification; LRD, light rocky desertification; MRD, moderate rocky desertification; SRD, severe rocky desertification.

**FIGURE 5:**
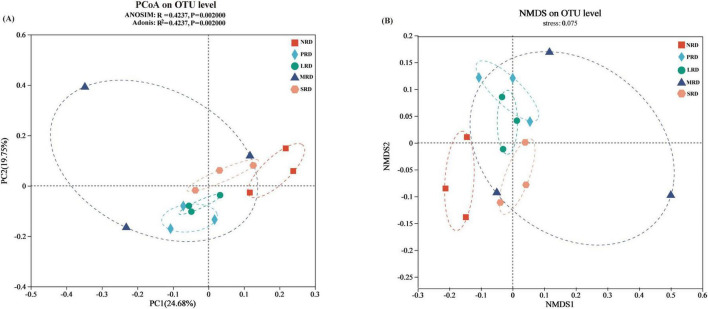
PCoA **(A)** and NMDS **(B)** analysis of soil bacterial communities under varying degrees of rocky desertification. NRD, nil rock desertification; PRD, potential rocky desertification; LRD, light rocky desertification; MRD, moderate rocky desertification; SRD, severe rocky desertification.

**FIGURE 6:**
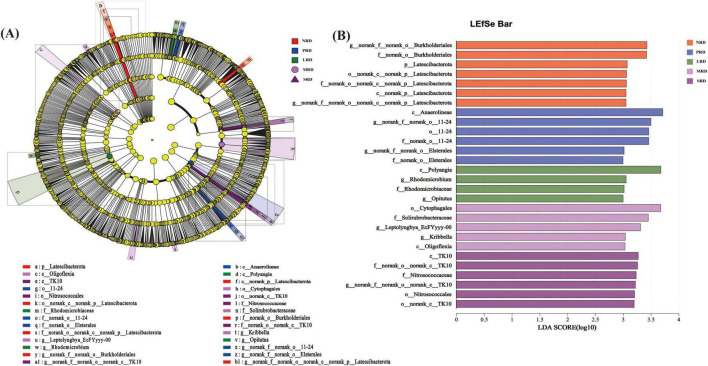
Cladogram of the bacterial lineages’ phylo-genetic distribution under varying degrees of rocky desertification **(A,B)**. NRD, nil rock desertification; PRD, potential rocky desertification; LRD, light rocky desertification; MRD, moderate rocky desertification; SRD, severe rocky desertification.

With the intensification of alpine grassland rocky desertification, the diversity of soil bacterial communities (Shannon, Simpson, Chao1 and ACE indexes) did not present a significant difference (*P* > 0.05), Shannon’s diversity indexes were highest in LRD, and Chao1’s richness indexes were also highest in LRD (Figure 4). Bray-Curtis distance, based on principal coordinate analysis (PCoA) and non-metric multidimensional scaling (NMDS), the bacterial communities are significantly different between the five levels of rocky desertification of alpine grasslands (ANOSIM: *R* = 0.424, *p* = 0.002; Adonis: *R*^2^ = 0.402, *p* = 0.004) (Figure 5A), NMDS the stress of NMDS is less than 0.1, the graph has some explanatory significance (Figure 5B). The LEfSE analysis (Figure 6) revealed statistically significant differences between the five degrees of rocky desertification in the compositions of the bacterial communities, with an LDA threshold of 3.0. Additionally, 28 branches of the bacterial community displayed statistically significant differences between the various degrees of rocky desertification. In terms of the makeup of the soil bacterial community, Burkholderiales (from family to genus), Latescibacterota (from phylum to genus), and Elsterales (from family to genus) were shown to be significantly enriched in NRD, PRD, and NRD, respectively. Significant enrichment of Rhodomicrobium (from family to genus) was observed in LRD. In SRD, TK10 (from class to genus) was significantly enriched.

### 3.4 Correlation of soil bacterial communities with environmental factors

**FIGURE 7:**
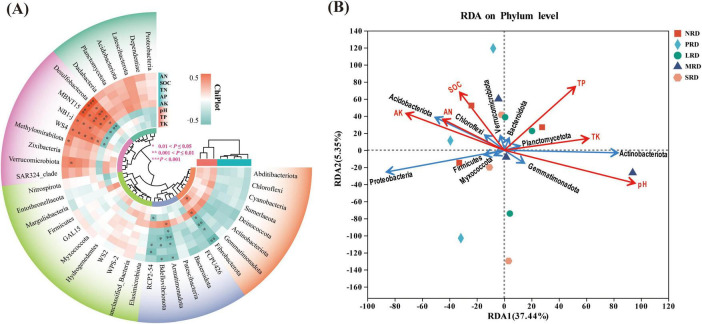
Heatmap analysis **(A)** and redundancy analysis (RDA) **(B)** of the bacterial communities with soil properties under varying degrees of rocky desertification. NRD, nil rock desertification; PRD, potential rocky desertification; LRD, light-rocky desertification; MRD, moderate-rocky desertification; SRD, severe rocky desertification.

**FIGURE 8:**
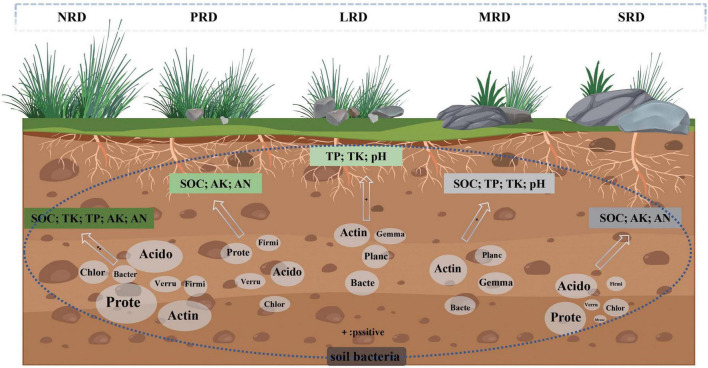
Relationships between the microbial populations and soil characteristics under varying degrees of rocky desertification. The abbreviation for soil bacteria is in the white circle. A large circle indicates a high degree of correlation. On the contrary, the correlation is small.

Redundancy analysis (RDA) and heatmap were used to examine the link between the soil bacterial community and environmental factors in the rocky desertification of alpine meadows at varying degrees (Figure 7). Through Spearman’s correlation calculation, environmental factors and species levels were clustered by averaging, and the top 30 species with abundance at the phylum level were selected for Heatmap correlation analysis. The results showed that Desulfobacterota, MBNT15, NB1-j, Methylomirabilota, Acidobacteriota and Verrucomicrobiota were densely distributed and close to each other and had a significant correlation with the environmental factors SOC, AN, TN, AP, and AK (Figure 7A). The study revealed significant correlations between soil variables (pH, SOC, TP, TK, AN, AK) and the soil bacterial community composition. Specifically, pH and TK showed positive correlations with Actinobacteriota abundance, whereas SOC, AN, and AK were positively correlated with Acidobacteriota. Notably, pH exhibited a negative correlation with Acidobacteriota. Additionally, other soil environmental factors also demonstrated significant associations with dominant bacterial taxa (Figure 7B). The results of the interaction between soil properties and bacterial communities showed that the correlation between soil nutrients and bacterial communities was strong under the mild rocky desertification gradient, and the correlation weakened with deeper evolution, and that the changes in the soil environment under the rocky desertification gradient succession had a significant effect on the composition of soil bacterial populations (Figure 8).

## 4 Discussion

In our study, microbial abundance and diversity did not show significant changes across the five levels of rocky desertification, and no clear or consistent trends were easily observed. This result contrasts with many studies on desertification’s effects on microbial communities, which typically report a significant decline in microbial diversity as desertification progresses (Zhang et al., 2016; Hu et al., 2017). For instance, studies in alpine grasslands on the Qinghai-Tibet Plateau have shown that desertification is closely linked to the reduction of microbial diversity, especially with reductions in soil organic carbon and nitrogen (Xue et al., 2019). However, in our study, microbial communities did not exhibit this consistent decline across different levels of rocky desertification. This may be attributed to the unique ecological features of the study area, especially the cold alpine environment and the specific form of rocky desertification observed. In this region, the thin soils and the underlying rocks and gravel may contribute to a smaller variation in microbial communities across desertification stages. Microbial community structure change was mainly related to the alkalinity of the soils in which they were found and their poor nutritional status in cold environments. Soil pH significantly affects soil bacterial communities in different types of soil ecosystems (Wang C. et al., 2019; Xiong et al., 2014). Microbial diversity was found to be highest when pH was close to neutral. Deviations from neutral pH led to environmental stress on microbial communities, resulting in reduced diversity due to selective pressures (Wang et al., 2017). Similar results have been reported in other extreme environments, such as the Arctic and high-altitude meadows, where microbial communities often show relative stability in response to environmental changes (Männistö et al., 2007). Additionally, the impact of rocky desertification may not be limited to microbial abundance and diversity alone but could also be influenced by other ecological factors such as soil moisture, temperature fluctuations, and soil structure (Zhao et al., 2019). Although we did not observe significant changes in microbial abundance and diversity across desertification levels, these factors could have a more profound effect on microbial function, community structure, and soil ecosystem services. Furthermore, studies suggest that certain microbial groups may exhibit strong adaptive traits in the early stages of desertification, helping maintain community diversity (Zhang et al., 2023). These microbes may adapt to changing soil conditions and nutrient limitations, allowing them to persist in degraded environments. In fact, some regions experiencing rocky desertification have shown that although microbial abundance decreased, key microbial functions persisted, highlighting the complex interactions between soil conditions and microbial communities (Marasco et al., 2023).

In general, positive succession refers to the improvement of soil properties over time, whereas reverse succession denotes soil degradation (Zhu et al., 2012). Studies indicate that soil nutrient content decreases as rocky desertification intensifies, creating a positive feedback loop with the rocky desertification process (Xie et al., 2015). Areas experiencing intense rocky desertification typically exhibit the lowest soil nutrient content and species diversity (Huang et al., 2012). However, some studies have shown that soil nutrients are not affected by increasing the degree of rocky desertification but improve after a first degradation trend (Sheng et al., 2015; Chen et al., 2018). Our results agreed with the former, showing that soil nutrients decrease with deepening rocky desertification. Among the eight properties measured in this study, the contents of TP and TK initially increased and then decreased with the intensification of rocky desertification. These indicators did not degrade consistently with increasing desertification but showed improvement after the initial degradation trend. With the deepening of rocky desertification, the contents of SOC, TN, AN, AP, and AK decreased overall. Notably, SOC and AP exhibited fluctuations with increases during the MRD stage, which could be attributed to factors such as rock weathering or nutrient redistribution. These findings suggest that SOC and AP may temporarily accumulate during the moderate stages of desertification but eventually decline as degradation progresses further. Overall, the results indicate that soil nutrient limitations become increasingly pronounced as rocky desertification intensifies.

The pH increased with increasing rocky desertification, and the pH of moderate rocky desertification soil was significantly higher than that of nil rocky desertification soil. Studies have shown that the pH value may be an indicator of rocky desertification in soil (Xie et al., 2015), which may be because in regions heavily affected by rocky desertification, extensive rock weathering leads to substantial rock dissolution, influencing the release of cations that can alter soil pH (Qi et al., 2018). Huang et al. (2021) observed that soil organic carbon (SOC) content in nil rocky desertification areas was significantly lower compared to rocky desertification areas. They also highlighted that rocky desertification areas, identified as “hot spots” of global greening (Xiao et al., 2021), have shown a notable enhancement in carbon sequestration capacity, which differs from our findings (Huang et al., 2021). Our findings indicated a decrease in soil organic carbon (SOC) with the increasing severity of rocky desertification grades, primarily attributable to higher rates of bedrock exposure in areas experiencing more severe rocky desertification. In comparison to the nil rocky desertification soil in southwest China, the soil of healthy alpine grasslands in Qinghai-Tibet is rich in organic carbon, and the limestone on the grasslands after rocky desertification has not yet decomposed organic matter. Furthermore, we found that soil TP and TK were higher in moderate and severe rocky desertification. These nutrients on the surface of exposed rocks were washed into the surrounding soil by rain or wind, resulting in increased soil nutrient content by exposed rocks.

The degradation of grassland into rocky desertification is a multifaceted process impacting above-ground vegetation coverage, biomass, diversity, and soil quality, subsequently influencing soil microbial communities (Fry et al., 2016; Pan et al., 2020). Cheng et al. (2016) observed significant changes in soil bacterial communities during the progression of rocky desertification, highlighting the critical role of soil characteristics in shaping microbial composition during the transition from alpine grasslands to rocky desertification. Several studies have consistently demonstrated that soil bacterial community structure is significantly influenced by environmental conditions, with soil factors playing a pivotal role (Chen et al., 2017; Saleem, 2015). In our study, Redundancy Analysis (RDA) highlighted pH as a key factor shaping soil bacterial communities across different vegetation restoration scenarios, consistent with findings by Fan et al. (2019) and Wang C. et al. (2019). The importance of soil pH in determining the composition of the soil bacterial community has been highlighted by a number of research (Griffiths et al., 2011; Zhang et al., 2022). The restricted growth tolerance and difficult development circumstances for many bacterial taxa in karst settings may be the cause of the high correlation found between pH and soil microbial community structure (Xue et al., 2017). In accordance with the findings of numerous research, we found that pH was negatively correlated with the abundance of dominating flora, including Acidobacteria and Ascomycetes. This suggests that pH is a significant indicator that affects the composition of soil microbial communities (Zhang et al., 2020; Song et al., 2022). In contrast to the other two genera, the actinomycetes group’s relative abundance grew dramatically as the rocky desertification process deepened. This suggests that actinomycetes are sensitive to soil pH and prefer neutral or alkaline conditions in which to thrive and survive (Dai et al., 2018; Li et al., 2019; Thomson et al., 2010).

Our study provides valuable insights that can inform practical strategies for mitigating rocky desertification. Specifically, understanding the microbial community structure and its relationship with soil properties enables us to identify key factors that need to be managed to halt or reverse desertification. Rocky desertification affects the chemical properties of the soil, particularly changes in pH and nutrient levels. Improving soil pH and organic matter levels can be enhanced through interactions with microbial communities. Based on this finding, soil amendments such as organic compost or biochar can be used to improve soil properties. Targeted vegetation restoration, particularly with drought-resistant and nutrient-efficient plants, can help stabilize soil properties and improve microbial diversity, thereby creating conditions that support both plant growth and microbial communities. Additionally, grazing disturbance, as a factor influencing microbial community dynamics in our study, can be managed through controlled grazing practices, such as rotational grazing, which help reduce soil compaction and hoof erosion and prevent overgrazing. Overgrazing often exacerbates desertification. These practices contribute to maintaining a balanced soil ecosystem, promoting vegetation growth, and enhancing soil fertility.

## 5 Conclusion

This study revealed changes in bacterial community composition and the relationship between soil bacterial community composition and soil environment variables in alpine grassland rocky desertification on the Qinghai-Tibet Plateau and its results improved our understanding of alpine rocky desertification microorganisms. The soil physiochemical properties differed significantly across different stages of alpine grassland rocky desertification, but there was no significant effect on the diversity of soil bacterial communities. The dominant phylum of all samples was Proteobacteria, Acidobacteria, Actinobacteria, and Chloroflexi. Twenty-eight biomarkers were found in the rocky desertification of alpine grasslands through a linear discriminate analysis (LDA) effect size analysis (LEfSe). The redundancy analysis (RDA) revealed that the composition of soil bacterial communities were mostly dependent on pH, AK, TP, and SOC in alpine grassland rocky desertification. Given these findings, it is essential to conduct further research to deepen our understanding of the processes driving rocky desertification in alpine grasslands and its broader ecological impacts on alpine meadow ecosystems. Additionally, based on the ecological dynamics identified in this study, implementing conservation measures such as controlled grazing and soil amendments will be crucial to mitigate the expansion of rocky desertification and restore ecosystem function.

## Data Availability

The achieved raw sequence reads were submitted to the NCBI Sequence Read Archive under the accession numbers PRJNA1198648.

## Footnotes

1
https://cloud.majorbio.com

2
https://huttenhower.sph.harvard.edu/lefse
